# Deleterious genetic variants in ciliopathy genes increase risk of ritodrine-induced cardiac and pulmonary side effects

**DOI:** 10.1186/s12920-018-0323-4

**Published:** 2018-01-24

**Authors:** Heewon Seo, Eun Jin Kwon, Young-Ah You, Yoomi Park, Byung Joo Min, Kyunghun Yoo, Han-Sung Hwang, Ju Han Kim, Young Ju Kim

**Affiliations:** 1 Seoul National University Biomedical Informatics (SNUBI), Div. of Biomedical Informatics, Seoul National University College of Medicine, Seoul, 03080 Korea; 2 Medical Research Institute, College of Medicine, Ewha Womans University, Seoul, 07985 Korea; 3 Department of Obstetrics and Gynecology, Konkuk University Medical Center, Konkuk University School of Medicine, Seoul, 05030 Korea; 4 Department of Obstetrics and Gynecology, College of Medicine, Ewha Womans University Mok Dong Hospital, Seoul, 07985 Korea

**Keywords:** Ritodrine, Pulmonary oedema, Whole-exome sequencing, Ciliopathy, Joubert syndrome

## Abstract

**Background:**

Ritodrine is a commonly used tocolytic to prevent preterm labour. However, it can cause unexpected serious adverse reactions, such as pulmonary oedema, pulmonary congestion, and tachycardia. It is unknown whether such adverse reactions are associated with pharmacogenomic variants in patients.

**Methods:**

Whole-exome sequencing of 13 subjects with serious ritodrine-induced cardiac and pulmonary side-effects was performed to identify causal genes and variants. The deleterious impact of nonsynonymous substitutions for all genes was computed and compared between cases (*n* = 13) and controls (*n* = 30). The significant genes were annotated with Gene Ontology (GO), and the associated disease terms were categorised into four functional classes for functional enrichment tests. To assess the impact of distributed rare variants in cases with side effects, we carried out rare variant association tests with a minor allele frequency ≤ 1% using the burden test, the sequence Kernel association test (SKAT), and optimised SKAT.

**Results:**

We identified 28 genes that showed significantly lower gene-wise deleteriousness scores in cases than in controls. Three of the identified genes*—CYP1A1*, *CYP8B1*, and *SERPINA7—*are pharmacokinetic genes. The significantly identified genes were categorized into four functional classes: ion binding, ATP binding, Ca^2+^-related, and ciliopathies-related. These four classes were significantly enriched with ciliary genes according to SYSCILIA Gold Standard genes (*P* < 0.01), thus representing ciliary genes. Furthermore, SKAT showed a marginal trend toward significance after Bonferroni correction with Joubert Syndrome ciliopathy genes (*P* = 0.05). With respect to the pharmacokinetic genes, rs1048943 (*CYP1A1*) and rs1804495 (*SERPINA7*) showed a significantly higher frequency in cases than controls, as determined by Fisher’s exact test (*P* < 0.05 and *P* < 0.01, respectively).

**Conclusions:**

Ritodrine-induced cardiac and pulmonary side effects may be associated with deleterious genetic variants in ciliary and pharmacokinetic genes.

## Background

Preterm birth (PTB: before 37 weeks gestation) is a major cause of neonatal mortality and morbidity, and can cause long-term health problems [1, 2]. Babies born prematurely are at an increased risk of cerebral palsy, respiratory illnesses, and intellectual disabilities [3]. The incidence of PTB has been reported to range from approximately 4% in Eastern Asia to 17% in the United States [4, 5]. Despite extensive research, advances in obstetrics care, and the development of pharmacological agents designed to reduce PTB, few effective PTB therapies are available.

Tocolytic agents are medications used to delay PTB and suppress uterine contractions. Beta2-adrenergic receptor agonists, such as ritodrine, are widely used tocolytic drugs that are effective for uterine relaxation [6]. However, ritodrine can cause serious adverse effects, such as pulmonary congestion, pulmonary oedema, dyspnoea, and tachycardia for the mother and the foetus [7–9]. In Korea, 13.1% of patients receiving ritodrine experienced side effects [2]. Ritodrine was withdrawn from use in the United States, and is used on a limited basis in Europe and Asia [7, 9]. To our knowledge, few studies have aimed to identify genetic polymorphisms associated with side effects related to ritodrine treatment as a tocolytic therapy to prevent PTB. The relationship between a mutation in calcium voltage-gated channel subunit alpha 1 C (*CACNA1C*) and ritodrine-induced side effects was recently reported [10].

Whole-exome sequencing (WES) is currently used to identify novel genetic variants that affect protein function [11]. WES is being applied to identify candidate genes in Mendelian disorders, common diseases, and cancer [12]. In addition, rare variants associated with complex diseases have been found by WES [13]. For example, rare variants of identified genes have been found to affect low-density lipoprotein cholesterol levels [14]. However, genetic polymorphisms that lead to ritodrine-induced cardiac and pulmonary side effects have not yet been identified, and the molecular mechanisms underlying the adverse effects of ritodrine remain unclear.

Thus, we investigated genes associated with the side effects of ritodrine using WES from 13 ritodrine-treated subjects with serious side effects in PTB.

## Methods

### Ethics statement

This research involving human subjects and their genomic data was approved by the Institutional Review Boards of Ewha Womans University Medical Center and Konkuk University Medical Center (IRB Nos. ECT 06–127-7 and KUH1040034). Written Informed consent was obtained from each subject prior to their participation.

### Patient and control samples for WES

Thirteen Korean pregnant women were analyzed using next-generation sequencing technology. Four subjects had pulmonary embolism, and nine subjects had a combination of tachycardia, palpitation, and/or dyspnoea. Two subjects were treated at Konkuk University Medical Center and the others were treated at Ewha Womans University Medical Center (Case, Table 1). For the control group, we selected exomes of 30 healthy Korean subjects (Control, 11 females and 19 males) from in-house data, which were provided by volunteers at the Division of Biomedical Informatics at Seoul National University (http://www.snubi.org/). Although the subjects had never been exposed to ritodrine, the exomes were sequenced using the same platform as the cases to minimize platform-specific biases. A total of 43 Koreans were recruited for WES analysis.

**Table 1:** Clinical characteristics of 13 pregnant women

Sample	Maternal age (years)	Gestational age at administration (weeks)	Side effect(s)
S1	30–39	31.4	Dyspnea
S2	30–39	30.2	Pulmonary edema, Pulmonary congestion
S3	30–39	34.0	Tachycardia, Dyspnea
S4	30–39	35.1	Dyspnea
S5	20–29	35.4	Pulmonary edema, Dyspnea
S6	30–39	36.5	Tachycardia, Dyspnea
S7	30–39	15.2	Tachycardia, Dyspnea
S8	20–29	33.5	Dyspnea
S9	40–49	27.6	Tachycardia, Dyspnea, Palpitation
S10	20–29	20.3	Tachycardia, Dyspnea, Headache, Sweating
S11	20–29	30.6	Dyspnea, Palpitation
S12	20–29	30.5	Pulmonary edema
S13	40–49	32.2	Pulmonary edema

### WES and variant calls

Genomic DNA extracted from peripheral blood cells was amplified to generate 175–250-base pair (bp) DNA fragments spanning the protein-coding regions of human genome DNA using the Ion AmpliSeq Exome Panel (Thermo Fisher Scientific, Waltham, MA, USA). Library construction was performed to load the DNA samples into the semiconductor chip using the Ion AmpliSeq Exome Library Kit Plus, covering 57,742,646 bp (1.85% of human genomic regions) as described in the manufacturer’s instructions (Thermo Fisher Scientific). The exon-enriched DNA libraries were sequenced using the Ion Proton platform following the manufacturer’s instructions (Thermo Fisher Scientific). All subjects were sequenced with the PI chip, which generated a mean depth of 70× (a sufficient depth to interrogate the exons for mutations). Sequence alignment and MAP files were generated with Torrent Suite (v4.4) software (Thermo Fisher Scientific), and variants were identified using the Genome Analysis Toolkit (GATK; v2.8) software using ‘HaplotypeCaller’ against the GRCh37 version of the human reference genome [15].

### Validation with genotyping assay

We validated 11 cases with side effects (out of the 13 cases with sufficient DNA) using an array-based high throughput method for 32 variants in 20 genes. In the single nucleotide polymorphism (SNP) type assay, 40 ng of genomic DNA flanking the SNP of interest was amplified by polymerase chain reaction (PCR) with a specific target amplification primer set. PCR was performed as described in the manufacturer’s instructions (Fluidigm, San Francisco, CA, USA). After amplification, the SNP type assay reaction was carried out according to the manufacturer’s instructions. SNP analysis was performed using Fluidigm SNP Genotyping Analysis software (ver. 4.0.1).

### Predicting the deleterious impact of variants

The Sorting Intolerant from Tolerant (SIFT) [16, 17] algorithm predicts the effect of a coding variant on protein function, which is displayed as a single measure based on the conservation and scores precomputed and distributed by the J. Craig Venter Institute (http://sift.jcvi.org/). The SIFT Human database that supports GRCh37 Ensembl release 63 (the latest version) was downloaded. In total, 33,248,232 coding DNA sequence variants with a SIFT score, ranging from 0 to 1, and 19,729 protein-coding genes listed in the SIFT Human database were analyzed. Combined Annotation Dependent Depletion v1.3 (CADD; http://cadd.gs.washington.edu/) [18] and Polymorphism Phenotyping v2.2.2 (PolyPhen2; http://genetics.bwh.harvard.edu/pph2/) [19] were also downloaded to predict the deleteriousness of the annotated variants on protein function.

### Aggregation of the impact of variants within genes

The gene deleteriousness score (*G*), defined as the geometric mean of the SIFT scores for the multitude of deleterious variants in a gene [20], was applied to estimate the aggregate impact of all deleterious variants in the genes. *G* aggregates the impact of deleterious variants by combining the probabilities of estimation of the likelihood that protein function was altered for each gene. Multiple deleterious variants of the same gene may synergistically impact protein function. We included only variants with a SIFT score < 0.7 as an input of the geometric mean and replaced 0 to 10^− 8^ for the *G* score calculation. We assigned a *G* score of 1 when no variants were reported in a given gene. A lower *G* score indicates a more damaged function of the gene at the protein level.

### Identification of significantly altered genes

**Fig. 1:**
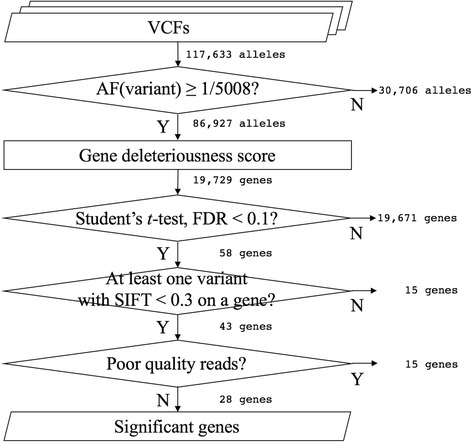
Workflow of data analysis for whole-exome sequencing. Workflow shows the steps used to select the significantly altered genes associated with ritodrine-induced side effects from 13 exomes. AF, allele frequency; FDR, false discovery rate; SIFT, sorting intolerant from tolerant

Among the 13 cases, a total of 558,091 variants were detected from 117,633 loci (Fig. 1). Initially, we included 86,927 loci with allele frequencies (AFs) ≥ 1/5008, as reported in the 1000 Genomes Project (T1GP; *n* = 2504), phase 3, with the assumption that variants in a highly curated public database would be less likely to contain errors [21, 22]. Next, we calculated a *G* score vector for all genes in each sample to assess the impact of deleterious variants on protein function. Student’s *t*-test was applied to identify altered function genes with deleterious variants by comparing the *G* score distribution between the case and control groups. We selected genes with at least one variant with SIFT < 0.3, which contributed to the lower *G* score. Lastly, to exclude likely false-positives, we reviewed each variant of the selected genes by manually inspecting all reads at all candidate loci in BAM files.

### Gene functional annotation and categorization

To interpret the biological relevance of the significant genes, we used DAVID v6.7 software to annotate Gene Ontology (GO) terms [23, 24]. In addition, we manually reviewed every gene to annotate known gene functions and associated diseases. We categorized the selected genes based on the annotations into four functional categories: ion binding, ATP binding, Ca^2+^-related, and ciliopathies. We obtained gene sets for the four categories; ion binding (GO: 0043167) and ATP binding (GO: 0005524) had corresponding GO terms that were exactly matched. Thus, we downloaded the gene sets annotated with the GO terms, which were comprised of 4386 and 1495 genes, respectively. However, the GO terms for the remaining two categories did not show an exact match. We searched for ‘calcium’ or ‘cilia’ as keywords to find the GO terms containing either of the keywords. As a result, we collected 1548 and 370 genes annotated to 312 and 42 GO terms for ‘Ca2 + −related’ and ‘ciliopathies’, respectively. We performed hypergeometric distribution tests with the four gene-sets to investigate the statistical significance of the selected genes.

### Ciliary genes and Joubert syndrome-related genes

Ciliary genes were extracted from The SYSCILIA Gold Standard Version 1, a high-confidence dataset that provides gene lists associated with ciliopathies (*n* = 303) [25]. In addition, we downloaded genes known to be involved in ciliopathy (*n* = 102) and Joubert and Meckel-Gruber syndromes (JBTMKS, *n* = 30) [26]. As the genes for Joubert syndrome (JBTS, *n* = 11) and Meckel-Gruber syndrome (MKS, *n* = 8) were grouped together in the JBTMKS category, we obtained genes for each syndrome from the Genetics Home Reference (GHR) [27, 28]. For each gene list, we carried out three rare-variant association tests: (1) the burden test, (2) sequence Kernel association test (SKAT), and (3) the optimised (SKAT-O) test using the SKAT package in R [13]. Only variants with a minor allele frequency (MAF) < 1% were selected for rare-variant association tests.

## Results

### Primary WES data analysis and genotyping assays

The Torrent Mapping and Alignment Program aligner in Torrent Suite generated, on average, 20 GB of BAM files per sample. Based on the GATK Best Practices guidelines, we modified a variant-calling pipeline to function with Ion Proton data and obtained an average of 41,819 ± 1976 [mean ± standard deviation (SD)] and 41,661 ± 1848 variants from the case and control groups (*P* = 0.81), respectively. Additionally, we confirmed that there was no significant difference in the number of variants between males and females in controls (*P* = 0.89). Next, we filtered out 30,706 variants that were not reported in the T1GP. We merged all variants that were called from individuals and annotated the SIFT score for each variant. Then, we calculated the *G* score for the genomic profile. The *G* score distributions were uniform across the different groups, showing a high and sharp peak near the highest score, which indicates a gene with normal function, a small peak near the lowest score, which indicates altered function, and a long plateau between the peaks, which indicates moderate function. Using the *G* score, we carried out Student’s *t*-test and found that the distributions of gene scores of 58 genes differed significantly at the significance level (false discovery rate, FDR < 0.1). We selected 43 genes that harboured deleterious and likely deleterious variants predicted by SIFT. For those selected genes, we concluded that the calls in 15 genes were false positive findings due to misalignment and/or off-target regions and that the remaining 28 genes were true positives. In summary, we identified 28 genes SIFT-predicted deleterious variants that were statistically significantly associated with ritodrine-induced cardiac and pulmonary side effects (Table 2). Additionally, all 32 variants that were predicted to be deleterious by either SIFT or CADD, among the 82 variants in the 28 significant genes, were successfully replicated using Fluidigm™ genotyping assays in 11 cases with sufficient DNA.

**Table 2:** Genes significantly associated with ritodrine-induced side effects

Gene symbol	Gene name	FDR	*P*	*G* (mean ± SD)		Gene functional category				
				Case, *(n* = 13)	Control, (*n* = 30)	ION	ATP	Ca^2+^	CP	PK
*AASDH*	Aminoadipate-Semialdehyde Dehydrogenase	0.019104	0.000041	0.16 ± 0.19	0.58 ± 0.44		✓	✓		
*ARMC9*	Armadillo Repeat Containing 9	0.025396	0.000057	0.06 ± 0.03	0.44 ± 0.46				✓	
*B9D2*	B9 Protein Domain 2	0.012597	0.000017	0.25 ± 0.33	0.82 ± 0.36				✓	
*BLK*	B lymphoid tyrosine kinase	0.007009	0.000004	0.16 ± 0.00	0.58 ± 0.43		✓			
*CARS2*	Cysteinyl-TRNA Synthetase 2, Mitochondrial (Putative)	0.068711	0.000227	0.23 ± 0.35	0.73 ± 0.43	✓	✓			
*CD1A*	CD1a Molecule	0.061968	0.000192	0.35 ± 0.37	0.86 ± 0.33					
*CSPG5*	Chondroitin Sulfate Proteoglycan 5 (Neuroglycan C)	0.075577	0.000336	0.24 ± 0.00	0.49 ± 0.36					
*CYP1A1*	Cytochrome P450, Family 1, Subfamily A, Polypeptide 1	0.065067	0.000209	0.03 ± 0.03	0.36 ± 0.46	✓		✓		✓
*CYP8B1*	Cytochrome P450, Family 8, Subfamily B, Polypeptide 1	0.012973	0.000020	0.45 ± 0.09	0.71 ± 0.26	✓				✓
*FAT4*	FAT Atypical Cadherin 4	0.092483	0.000539	0.23 ± 0.10	0.43 ± 0.28	✓		✓		
*FUT6*	Fucosyltransferase 6 (Alpha (1,3) Fucosyltransferase)	0.051144	0.000147	0.14 ± 0.04	0.46 ± 0.42					
*GALNT10*	Polypeptide N-Acetylgalactosaminyltransferase 10	0.092483	0.000549	0.35 ± 0.21	0.66 ± 0.35	✓				
*HHATL*	Hedgehog Acyltransferase-Like	0.076828	0.000369	0.27 ± 0.11	0.50 ± 0.30					
*IFT74*	Intraflagellar Transport 74	0.011408	0.000014	0.25 ± 0.10	0.56 ± 0.32				✓	
*ICE1*	Interactor Of Little Elongation Complex ELL Subunit 1	0.014088	0.000026	0.49 ± 0.08	0.70 ± 0.23					
*KNDC1*	Kinase Non-Catalytic C-Lobe Domain (KIND) Containing 1	0.099453	0.000617	0.34 ± 0.03	0.53 ± 0.29					
*NKAIN3*	Na+/K+ Transporting ATPase Interacting 3	0.014088	0.000026	0.21 ± 0.08	0.56 ± 0.40			✓		
*OR6B1*	Olfactory Receptor, Family 6, Subfamily B, Member 1	0.092483	0.000562	0.28 ± 0.02	0.50 ± 0.33					
*PSMD9*	Proteasome (Prosome, Macropain) 26S Subunit, Non-ATPase, 9	0.092483	0.000511	0.43 ± 0.40	0.91 ± 0.25					
*PXT1*	Peroxisomal, Testis Specific 1	0.075577	0.000336	0.02 ± 0.00	0.35 ± 0.47					
*RBBP8NL*	RBBP8 N-Terminal Like	0.092483	0.000564	0.21 ± 0.25	0.58 ± 0.43					
*RSPH3*	Radial Spoke 3 Homolog (Chlamydomonas)	0.012770	0.000018	0.27 ± 0.07	0.61 ± 0.37				✓	
*SERPINA7*	Serpin Peptidase Inhibitor, Clade A (Alpha-1 Antiproteinase, Antitrypsin), Member 7	0.077599	0.000400	0.31 ± 0.48	0.90 ± 0.30					✓
*SLC12A7*	Solute Carrier Family 12 (Potassium/Chloride Transporter), Member 7	0.051144	0.000148	0.36 ± 0.04	0.60 ± 0.31	✓				
*SPTA1*	Spectrin, Alpha, Erythrocytic 1	0.008266	0.000007	0.32 ± 0.08	0.49 ± 0.15	✓		✓		
*TNKS*	Tankyrase, TRF1-Interacting Ankyrin-Related ADP-Ribose Polymerase	0.077599	0.000401	0.38 ± 0.19	0.68 ± 0.33	✓				
*ZDHHC12*	Zinc Finger, DHHC-Type Containing 12	0.075577	0.000332	0.17 ± 0.15	0.41 ± 0.27	✓				
*ZNF273*	Zinc Finger Protein 273	0.092483	0.000527	0.10 ± 0.05	0.38 ± 0.42	✓				

*FDR* false discovery rate, *G* gene deleteriousness score, *ION* ion binding, *ATP* adenosine triphosphate binding, *Ca*^*2+*^ calcium-related, *CP* ciliopathy, *PK* pharmacokinetics–relate

### Genes associated with ritodrine-induced side effects

The hypergeometric test failed to identify a significantly enriched GO term, perhaps due to the small number of significant genes obtained. However, the hypergeometric distribution test revealed that the 28 genes showed significant enrichment with the SYSCILIA Gold Standard genes (*P* = 0.009). SYSCILIA is a multi-national consortium for a biology systems approach to dissect cilia function and its disruption in human genetic disease, and the SYSCILIA Gold Standard contains highly curated ciliary and ciliopathy genes. Even after lowering the definition stringency of deleterious genes between cases and controls from FDR < 0.01 to *P* < 0.01 and *P* < 0.05, the statistical significance of the SYSCILIA Gold Standard gene enrichment remained robust and sustained (*P*_0.01_ = 0.047 and *P*_0.05_ = 0.001, respectively). As shown in Table 2, the 28 significant genes (FDR < 0.1) were categorized into four functional classes: ion binding, ATP binding, Ca^2+^-related, and ciliopathies.

### Analysis of ciliopathy genes and their subsets

The SKAT rare variant association test between the case (*n* = 13) and control (*n* = 30) groups showed marginal significance for JBTMKS after Bonferroni correction (*P* = 0.1054, Table 3) from the Invitae. Since JBTMKS harbour both JBTS- and MKS-related genes, we further evaluated the association signals for JBTS and MKS separately using GHR. Only JBTS genes showed a marginal trend toward significance, whereas MKS genes did not. This result suggests that genetic variations identified in patients with serious ritodrine-induced cardiac and pulmonary side effects may be associated with JBTS. Table 4 presents the rare (MAF < 0.01) and deleterious (predicted to be deleterious by at least one Bioinformatics tools: SIFT, CADD, or PolyPhen2) variants in JBTS genes (*n* = 11) in patients with serious ritodrine-induced side effects. Of these, four genes (*AHI1*, *ARL13B*, *KIF7*, and *RPGRIP1L*) exhibited five heterozygous mutations, but no homozygous mutations. Pulmonary embolism, considered to be the most serious side effect induced by ritodrine, was observed in four patients (SN1923, SN3230, SN8592 and SN9899). Rs193219215 on ADP ribosylation factor-like GTPase 13B (*ARL13B*), which interacts with Intraflagellar transport 74 (*IFT74*) [29], was found in two cases with pulmonary embolism (SN3230 and SN8592). This nonsynonymous variant, predicted to be deleterious by CADD, is a rare variant (MAF < 0.001) that has been found only in East Asian (EAS) populations according to T1GP. Rs146925098 on RPGRIP1 like (*RPGRIP1L*) was found in one case with pulmonary embolism (SN3230). SN1923 and SN9899 cases with pulmonary embolism exhibited none of the above variants.

**Table 3:** Rare-variant association tests for genes associated with ciliopathies and subsets

Database	Gene list (No. of genes)	Bonferroni corrected *P*			Reference
		Burden	SKAT	SKAT-O	
Invitae	Ciliopathies (102)	1.00000	1.00000	1.00000	[26]
	Joubert and Meckel-Gruber Syndromes (JBTMKS; 30)	0.39528	0.10536	0.19548	[26]
GHR	Joubert Syndrome (JBTS; 11)	0.09908	0.05076	0.08020	[27]
	Meckel Syndrome (MKS; 8)	1.00000	1.00000	1.00000	[28]

*GHR* Genetics home references, *SKAT* sequence kernel association test

**Table 4:** Distribution of rare and deleterious variants on four Joubert Syndrome genes from GHR in 13 cases with ritodrine-induced serious cardiac and pulmonary side effects

Gene Symbol	rsID	Variant deleteriousness score			MAF	SN1275	SN1923	SN2931	SN2956	**SN3230**	SN3828	SN4181	SN4211	SN4407	SN5021	SN5209	SN8592	SN9899
		SIFT	CADD	PolyPhen2														
*AHI1*	rs148000791	0.25	23.6	NA	0.0077	HET	WT	HET	HET	WT	WT	WT	WT	WT	WT	WT	HET	WT
*ARL13B*	rs193219215	0.12	20.9	0.41	0.0008	WT	WT	WT	WT	HET	WT	WT	WT	WT	WT	WT	HET	WT
*KIF7*	rs546772749	0.01	9.2	0.01	0.0002	HET	WT	HET	HET	HET	WT	WT	WT	HET	HET	WT	WT	WT
	rs536773143	0.16	15.4	0.89	0.0002	WT	WT	WT	WT	WT	WT	WT	WT	WT	WT	WT	HET	WT
*RPGRIP1L*	rs146925098	0	19.7	0.99	0.0002	WT	WT	WT	WT	HET	WT	WT	WT	WT	WT	WT	WT	WT

*SIFT* Sorting Intolerant from Tolerant, *CADD* Combined Annotation Dependent Depletion, *PolyPhen2* Polymorphism Phenotyping v2.2.2, *AF* allele frequency, *HET* heterozygous, *WT* wild type, *AHI1* Abelson Helper Integration Site 1, *ARL13B* ADP Ribosylation Factor Like GTPase 13B, *KIF7* Kinesin Family Member 7, *RPGRIP1L* RPGRIP1 Like

### Drug metabolism-related genes

Two cytochrome P450 genes [family 1, subfamily A, polypeptide 1 (*CYP1A1*) and family 8, subfamily B, polypeptide 1 (*CYP8B1*)] and serpin family A member 7 (*SERPINA7*), which are involved in drug metabolism phases I and II, respectively, exhibited significantly lower G scores in the case group compared to those in the control group. Rs1048943 on *CYP1A1* was found in nine patients including two patients with pulmonary embolism (SN1932 and SN9899), and the carrier frequency in cases was significantly higher than in controls using Fisher’s exact test (*P* = 0.046, Table 5). The carrier frequency of rs143070677 on *CYP1A1* was significantly higher in cases than in the EAS population (*P* = 0.050), whereas it was not significant compared to the control group (*P* = 0.518). This allele was found in one patient with pulmonary embolism (SN3230) and is a singleton in T1GP (MAF < 0.0002). Rs4646422 on *CYP1A1* was found in four patients including one patient with pulmonary embolism (SN8592). In summary, either one of the two functional variants of *CYP1A1* was found in all patients with pulmonary embolism. These alleles are East Asian-specific, in that their frequencies are relatively higher in EAS than in other populations. Although a patient with rs202192572 on *CYP8B1* did not have pulmonary embolism, this is also a rare East Asian-specific variant that was found in only five EAS subjects in T1GP. The carrier frequency of rs1804495 on *SERPINA7* was significantly higher in cases than in control groups (*P*_Control_ < 0.001, *P*_EAS_ = 0.018, and *P*_T1GP_ = 0.001), and nine patients carried this allele including two with pulmonary embolism. Rs1804495 was also over-represented in the EAS population.

**Table 5:** Carrier frequencies and Fisher’s exact test results of variants on the drug metabolism-related genes

Gene Symbol	rsID	Variant deleteriousness score			Case, *n* = 13	Control, *n* = 30		EAS, *n* = 504		T1GP, *n* = 2504	
		SIFT	CADD	PolyPhen2		Freq	*P*	Freq	*P*	Freq	*P*
*CYP1A1*	rs1048943	0.01	21.9	0.49	9 (69%)	10 (33%)	0.0457	221 (44%)	0.0902	568 (23%)	0.0005
	rs143070677	0.03	24.4	0.51	1 (8%)	1 (3%)	0.5183	1 (0.2%)	0.0497	1 (0.04%)	0.0103
	rs4646422	0.08	24.0	0.19	4 (31%)	12 (40%)	0.7349	111 (22%)	0.4986	116 (5%)	0.0025
*CYP8B1*	rs202192572	0.04	23.9	0.05	1 (8%)	0 (0%)	0.3023	5 (1%)	0.1423	5 (0.2%)	0.0306
*SERPINA7*	rs1804495	0.01	23.8	0.46	9 (69%)	3 (10%)	0.0002	179 (36%)	0.0180	623 (25%)	0.0010

*Freq* carrier frequency, *SIFT* Sorting Intolerant from Tolerant, *CADD* Combined Annotation Dependent Depletion, *PolyPhen2* Polymorphism Phenotyping v2.2.2, *EAS* East Asian in the 1000 Genomes Project, *T1GP* The 1000 Genomes Project, *CYP1A1* cytochrome P450, family 1, subfamily A, polypeptide 1, *CYP8B1* cytochrome P450, family 8, subfamily B, polypeptide 1, *SERPINA7* serpin peptidase inhibitor, clade A (alpha-1 antiproteinase, antitrypsin), member 7

## Discussion

Little is known about the physiological mechanism of ritodrine; *ADRB2* is the only known target. Here, we analyzed the whole-exomes of 13 cases with serious side effects and identified 28 genes that increase the risk of ritodrine-induced cardiac and pulmonary side effects. To the best of our knowledge, this study was the first analysis using next-generation sequencing data to identify associations between ritodrine side effects and genomic variants. Using the *G* scoring method, ion binding, ATP binding, Ca^2+^-related, and ciliopathy genes were found to be significantly altered in protein function. In particular, ion binding, ATP binding, and Ca^2+^-related genes are important and well-known for their roles in tocolysis. In addition, rare variants in cases were associated with JBTS genes using RVATs. Two out of four pulmonary embolism cases carried at least one rare and deleterious variant on JBTS genes. Consequently, we speculate that rare variants on JBTS genes may contribute to the ritodrine-induced side effects related to muscle flexibility.

In addition to identifying ciliopathy genes, we detected ion- and ATP-binding, and calcium-related, genes that were significantly associated with ritodrine-induced side effects. These genes have not been reported as being disease-related genes and are not members of a pathway, implying that they may not play a key role in pathogenicity. The results suggest that, the significant genes may be important in drug responses; however, further investigations are required. The molecular functions of these genes have been well-studied. Calcium, ATP and inorganic phosphate play key roles in the physiological functions of proteins, including muscle contraction [30]. In particular, myometrium contraction is affected by increased calcium levels and activation of the myosin light chain kinase via phosphorylation [31]. Ritodrine causes relaxation of smooth muscles in the uterus while stimulating cardiac muscles [31, 32]. In addition, mutations in sulfotransferase 1A3 were determined to affect the metabolic activity of ritodrine [33]. Collectively, we speculate that genetic predisposition related to ion- and ATP-binding and calcium plays an important role in the side effects of ritodrine treatment of PTB. An association between rs10774053 in *CACNA1C* and ritodrine side effects was reported recently [10]; however, this genetic association was not replicated in this study as no significant difference (*P* = 0.1962) was detected in *CACNA1C*; *G* score (mean ± SD) between the case and the control groups (0.28 ± 0.2 and 0.35 ± 0.3, respectively). However, they selected five SNPs in *CACNA1C*, including three and two variants in intron and exon regions, respectively. The two exonic variants are predicted to be benign according to SIFT, and a recessive model was applied for statistical tests.

The distribution of deleterious variants of pharmacokinetic genes differs markedly across ethnic groups. Two rare Asian-specific functional alleles (rs143070677 and rs202192572) were reported only in an EAS population with MAF < 0.001. Rs1048943, rs4646422, and rs1804495 are common alleles (MAF > 0.05), and MAFs in EAS are relatively higher than those in other ethnic groups. *CYP1A1* and *CYP8B1*, which are xenobiotic enzymes, are involved in the metabolism of drug and toxicants [34]. Elevated *CYP1A1* activity causes the formation of DNA adducts in pulmonary tissue and cellular damage [35, 36]. A particularly interesting finding was that at least one of the functional variants of *CYP1A1* was identified in pulmonary embolism cases in this study. An association of *CYP1A1* polymorphisms with an increase in *CYP1A1* activity has been confirmed by functional studies [37, 38]. In particular, the catalytic activity in oestrogen metabolism was significantly higher in those with rs1048943 than wild-type [39]. The tocolytic effect of ritodrine was enhanced by adding natural progesterone in pregnant women [40]. A more recent study revealed the loss of a transcription factor binding site at Sp7 due to rs1048943 at exon 7 of *CYP1A1*; this variant was predicted to be deleterious by SIFT and CADD [41]. We also identified rare variants in drug metabolism-related genes with a relatively higher frequency in Asian subjects, despite the small sample size. Therefore, overrepresented and/or significantly higher deleterious variants in drug metabolism genes may also increase the risk of ritodrine-induced side effects, such as pulmonary embolism, in the Korean population.

## Conclusions

Using WES, this study identified rare deleterious variants associated with ritodrine-induced serious cardiac and pulmonary side effects in Korean preterm labour subjects. Most importantly, rare variants on ciliopathy genes were demonstrated to be significantly associated with JBTS. Asian-specific rare and common variants related to the pharmacokinetics of ritodrine may elicit serious cardiac and pulmonary side effects. Further studies are needed to validate the rare variants in a larger cohort for replication, and to elucidate the role of these variants in the molecular mechanisms of the side effects.
